# The Effect of Nano-Titanium Dioxide on Limb Bud Development of NMRI Mouse Embryo *In Vivo*

**DOI:** 10.22074/cellj.2016.3734

**Published:** 2015-07-11

**Authors:** Kazem Parivar, Nasim Hayati Rudbari, Ramazan Khanbabaee, Mahya Khaleghi

**Affiliations:** 1. Department of Biology, Faculty of Science, Islamic Azad University, Science and Research Branch, Tehran, Iran; 2. Department of Biology, Faculty of Science, Islamic Azad University, Ghaemshahr, Mazandaran, Iran

**Keywords:** Titanium Dioxide, Nanoparticles, Limb Bud, Chondrogenesis

## Abstract

**Objective:**

There is a wide application of titanium dioxide (TiO_2_) nanoparticles (NPs) in industry. These particles are used in various products, and they also has biological effects on cells and organs through direct contact.

**Materials and Methods:**

In this experimental research, the effect of TiO_2_ on chondrogenesis of forelimb buds of mice embryos was assessed in *in vivo* condition. Concentrations of 30, 150 and 500 mg/kg body weight (BW) TiO_2_ NPs (20 nm size) dissolved in distilled water were injected intraperitoneally to Naval Medical Research Institute (NMRI) mice on day 11.5 of gestation. On day 15, limb buds were amputated from the embryos and skeletogeneis of limb buds were studied.

**Results:**

TiO_2_ NPs caused the significant changes in chondrocytes in the following developmental stages: resting, proliferating, hypertrophy, degenerating, perichondrium and mesenchymal cells. Decreased number of mesenchymal cells and increased level of chondrocytes were observed after the injection of different concentrations of TiO_2_, which proves the unpredictable effects of TiO_2_ on limb buds.

**Conclusion:**

Results of the present study showed TiO_2_ NPs accelerated the chondrogenesis of limb buds, but further studies are recommended to predict TiO_2_ toxicity effects on organogenesis.

## Introduction

Titanium dioxide (TiO_2_) is widely used as an industrial nanomaterial. The use of TiO_2_ nanoparticles (NPs) as an additives in pharmaceutical and food industries as well as in various products, including coatings, ceramics, anti-fouling paints, cosmetics and sunscreens, has gained increasing attention in past few years (1, 2). The unique physicochemical characteristics of NPs, such as high reactivity, small size, and large surface area per mass, have raised great concerns on the adverse effects of TiO_2_ NPs on ecological system and human health (3-5). Due to its widespread use, humans are increasingly exposed to TiO_2_ NPs material. The respiratory tract and skin are the main exposure areas. It has been also demonstrated that TiO_2_ NPs changes phagocytic activity in cultured macro-phages and the integrity of the cell membrane (6). TiO_2_ occurs primarily in the forms of the minerals rutile, anatase and brookite (7, 8). Therefore, we aimed to evaluate the effects of TiO_2_ NPs on chondrogenesis of forelimbs skeletons of mouse embryo *in vivo*.

## Materials and Methods

### Animals and treatment

In this experimental research, we studied the effect of TiO_2_ on limb bud of mice. This project was carried out at the Animal Biology Laboratory of Islamic Azad University, Science and Research Branch, Tehran, Iran. Naval Medical Research Institute (NMRI) mice (5 males, 20 females, 30 ± 5 g) were purchased from the Pasture Institute, Tehran, Iran. Mice were housed in an animal room at 24 ± 2˚C with a 12-hour light/dark cycle for five days, before starting the experiment. Food and water were provided and copulations were set up. Day one of vaginal plug observation was determined as day 0 of gestation. After 11.5 days, the body weight of animals were weighed and randomly divided into 5 groups (n=3 per group). Three experimental groups were injected intraperitoneally with size 20 nm TiO_2_ NPs at concentrations of 30, 150 and 500 mg/kg body weight (BW), a sham group received an injection of 1ml distilled water, and non-injected group was assigned as control group. Four days later, the mice were sacrificed after being anesthetized by ether. All procedures used in animal experiments were in compliance with the Ethics Committee of Science and Research Branch of Islamic Azad University. Embryos were excised from uterus and amniotic membrane, and then both right and left forelimbs were carefully amputated, rinsed with Hank’ balanced salt solution (HBSS, Merck, Germany), and prepared for histological and morphological studies.

### Histological methods

**Fig.1:**
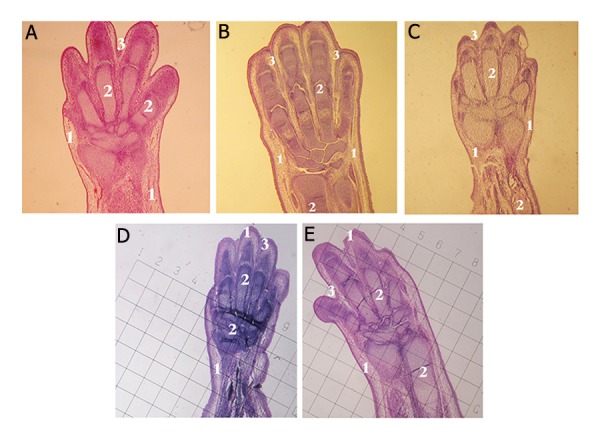
Photomicrograph of limb bud tissues on day 15 of development. Samples of each group were fixed in Bouin’s fluid, embedded in paraffin, serially sectioned (5-μm thick) and stained using hematoxylin-eosin (HE) method. A. Control ×40, B. Sham ×40, C. Concentration of 30, D. 150 and E. 500 ×40. 1; Mesenchymal cells, 2; Chondrosytes and 3; Finger grooves.

**Fig.2:**
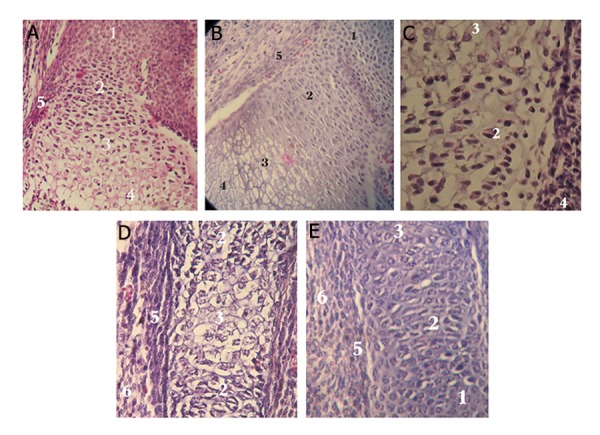
Photomicrograph of limb bud tissues on day 15 of development. A. Chodrosytes cells in control sample ×100, B. Condrocytes in sham sample ×100, exposed to distilled water. Chondrocytes in experimental samples ×400, C. They were exposed to titanium dioxide (TiO₂) nanoparticles (NPs) at concentrations of 30, D. 150 and E. 500 mg/kg body weight (BW) on day 11¬ of development. 1; Resting chondrosytes, 2; Proliferating chondrosytes, 3; Hyperterophic chondrosytes, 4; Degenerating chondrosytes, 5; Perichondrium cells and 6; Mesenchymal cells.

The tissues were immediately fixed in Bouin’solution (Merck, Germany), for 2 hours. Briefly the samples were embedded in paraffi n blocks, serially sectiond into 5-μm in thickness and placed onto the glass slides. After hematoxylin-eosin (HE) staining, the slides were observed using an optical microscope (Nikon, USA) (Fig.1). The following measurments were then perfomred in each groups: length of finger-palm (region 1), length of wrist-forearm (region 2) and arm-forearm (region 3), using scaled graticule. Furthermore number of mesenchymal, perichondrial cells, resting, proliferating, hypertrophic and degenerating chondrocytes cells were counted in 3 mentioned region from 6 fields of microscope views in each groups. The mesenchymal cells contains a large, round nucleus, which is surrounded by long cytoplasm. The perichondrium is a sheath of dense connective tissue that surrounds cartilage in most places. The resting zone consists typical round chondrocytes. In the proliferative zone, oval chondrocytes begin to divide rapidly and form columns of stacked cells parallel to the long axis of the bone. The hypertrophic zone contains swollen chondrocytes appeatring after proliferating chondrocytes. The degenerative zone contains disintegrated swollen chondrocytes (Fig.2).

### Statistical analysis

The mean values of forelimbs parameters were calculated from six sections per slide in each group (n=6). All data were conducted using the SPSS (SPSS Inc., Chicago, IL, USA) software. Oneway ANOVA was used to compare the differences among means belonging to multi-group data. All values were calculated from standard errors of means and statistical significance was considered at level of P≤0.05.

## Results

### Morphometric study

**Fig.3:**
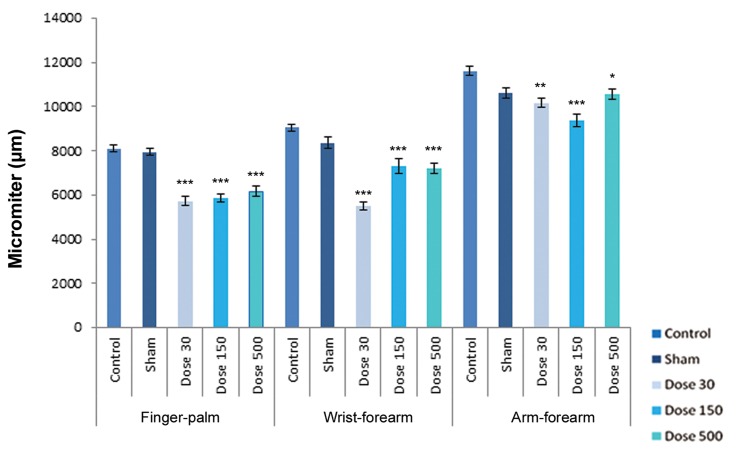
Histograms of length of regions in forelimb buds on day 15 of development. Region 1 belonging to finger-palm, region 2 belonging to wrist-forearm and region 3 belonging to arm-forearm. Data are presented as means ± SEM. *; P <0.05, **; P <0.01 and ***; P <0.001.

In the experimental groups, the limb bud tissue had no abnormal pathological changes compared with the control. Abortion percentage in experimental groups was 8%. Morphological differences were observed in length of forelimb buds and the experimental samples showed a significant decrease in length of finger-palm (region 1), length of wrist-forearm (region 2), and arm-forearm (region 3), as compared with the control and sham groups (Fig.3).

### Histological examinations

**Fig.4:**
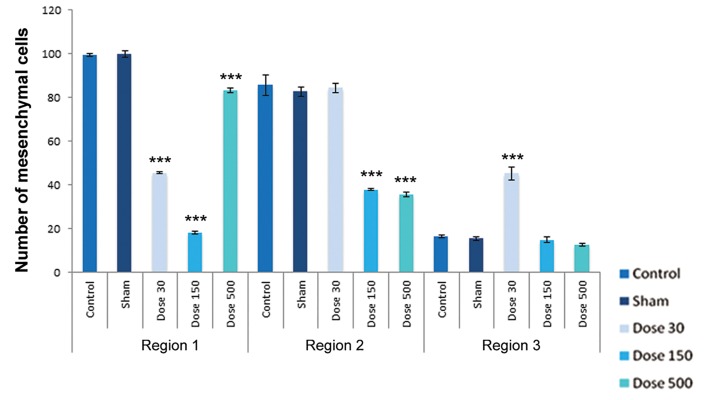
Histograms of number of mesenchymal cells in control, sham and experimental groups on day 15 of development. Region 1 belonging to finger-palm, region 2 belonging to wrist-forearm and region 3 belonging to arm-forearm region. Data are presented as means ± SEM. *; P<0.05, **; P <0.01 and ***; P<0.001.

**Fig.5:**
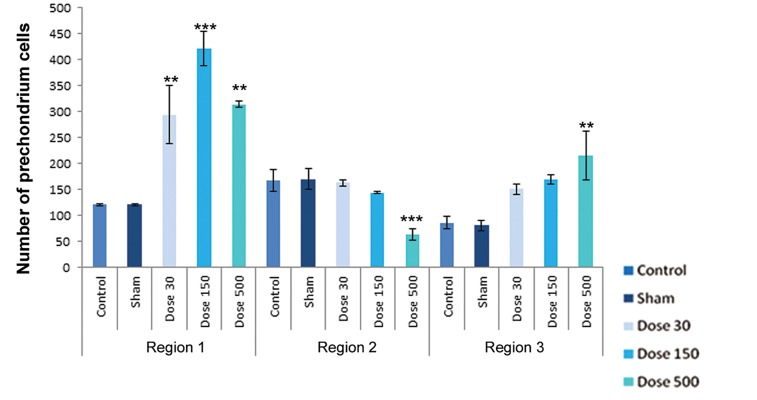
Histograms of number of perichondrium cells in control, sham and experimental groups on day 15 of development. Region 1 belonging to finger-palm, region 2 belonging to wrist-forearm and region 3 belonging to arm-forearm. Data are presented as means ± SEM. *; P<0.05, **; P<0.01 and ***; P<0.001.

**Fig.6:**
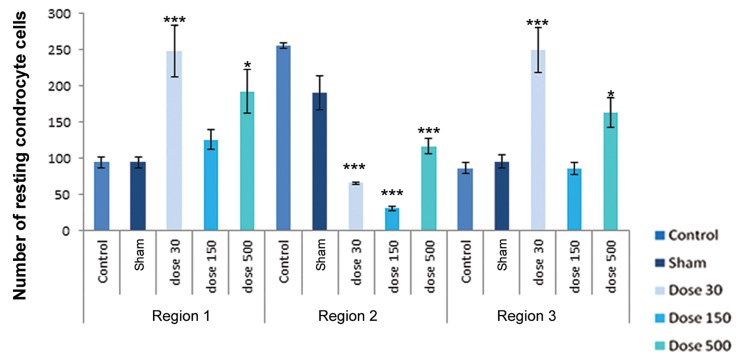
Histograms of number of resting chondrocytes in control, sham and experimental groups on day 15 of development. Region 1 belonging to finger-palm region, region 2 belonging to wrist-forearm region and region 3 belonging to arm-forearm region. Data are presented as means ±SEM. *; P <0.05, **; P<0.01 and ***; P<0.001.

**Fig.7:**
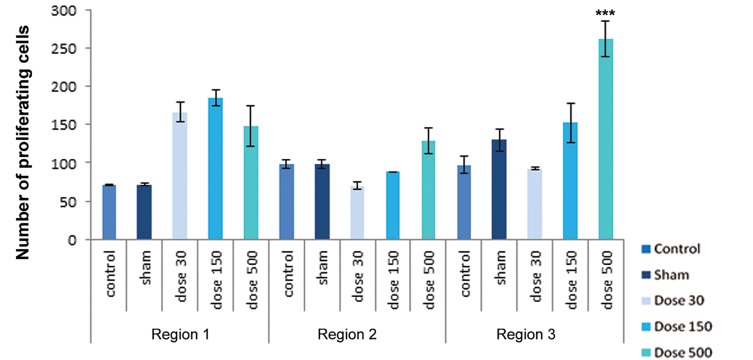
Histograms of number of proliferating chondrocytes in control, sham and experimental groups on day 15 of development. Region 1 belonging to finger-palm region, region 2 belonging to wrist-forearm region and region 3 belonging to arm-forearm region. Data are presented as means ± SEM. *; P<0.05, **; P<0.01 and ***; P<0.001.

**Fig.8:**
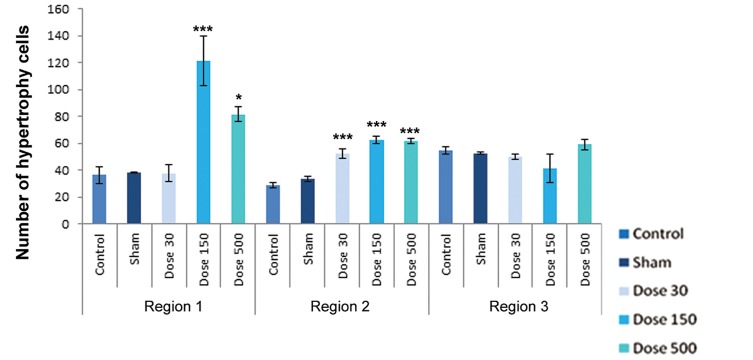
Histograms of number of hypertrophic chondrocytes in control, sham and experimental groups on day 15 of development. Region 1 belonging to finger-palm, region 2 belonging to wrist-forearm and region 3 belonging to arm-forearm. Data are presented as means ± SEM. *; P<0.05, **; P<0.01 and ***; P<0.001.

**Fig.9:**
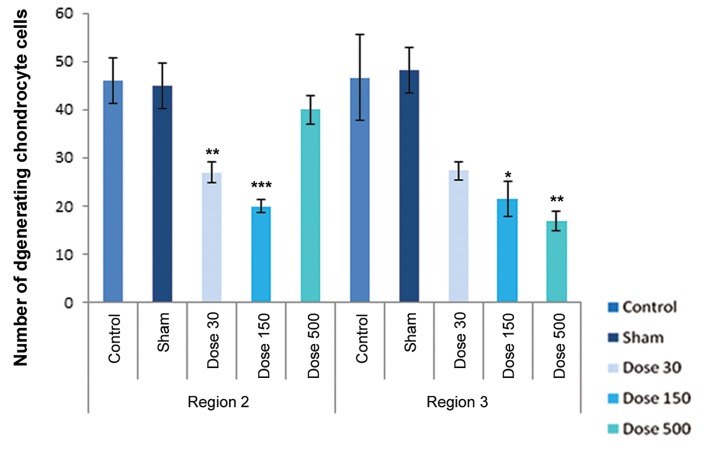
Histograms of number of degenerating chondrocytes in control, sham and experimental groups on day 15 of development. Region 1 belonging to finger-palm, region 2 belonging to wrist-forearm and region 3 belonging to arm-forearm. Data are presented as means ± SEM. *; P<0.05, **; P<0.01 and ***; P<0.001.

Comparison of the forelimb buds sections exposured to different concentrations of TiO_2_ NPs confirmed significant changes in cell proliferation and differentiation. Figures 4-9 shows the change of chondrocyte numbers in forelimb buds.

In finger-wrist (region 1), mesenchymal cell counts showed a significant decrease in experimental groups campared with sham and control groups, but perichondrial cells showed a signiﬁcant increase in comparison with the sham and control groups. Resting chondrocytes counts in this region showed an increase as campared with sham and control groups and this increase was significant in the 30 mg/kg TiO_2_ treatment. The number of proliferating chondrocytes showed no signiﬁcant changes in this region in all treatments.

Hypertrophic chondrocytes showed a signifi cant increase in both 150 and 500 mg/kg TiO_2_-treated groups, whereas there were no significant differences in 30 mg/kg TiO_2_ treatment in comparison with the sham and control groups. Degenerating chondrocytes were observed in wrist-forearm (region 2) and arm-forearm (region 3).

In wrist-forearm (region 2), mesenchymal cell counts showed a signifi cant decrease in 150 and 500 mg/kg TiO_2_-treated groups campared with the sham and control groups, indicating there were no signifi cant differences in 30 mg/kg TiO_2_ treatment. Perichondrial cells showed no signifi cant changes in comparison with the sham and control groups, but a signifi cant decrease by exposure to 500 mg/kg TiO_2_ were observed. Resting chondrocytes in this region showed a signifi cant decrease campared with the sham and control samples. Proliferating chondrocytes revealed no signifi cant changes campared with the sham and control groups. Hypertrophic chondrocytes counts in all experimental groups showed a signifi cant increase in campared with the sham and control groups. The number of degenerating chondrocytes were reduced by exposure to 500 mg/kg TiO_2_, but this decrease was not signifi cant; howerer, the number of cells by exposure to concentrations of 30 and 150 mg/kg TiO_2_ were signifi cantly decreased.

In arm-forearm (region 3), mesenchymal cell counts were signifi cantly higher in 30 mg/kg TiO_2_ treatment than exposured to concentrations of 150 and 500 mg/kg TiO_2_, showing no signifi cant changes campared with the sham and control groups. Perichondrial cells showed a significant increase in 500 mg/kg TiO_2_ treatment in comparison with the sham and control samples. Resting chondrocytes counts showed a significant increases in 30 and 500 mg/kg TiO_2_ treatments campared with the sham and control groups. Proliferating chondrocytes showed no significant changes in 30 and 150 mg/kg TiO_2_ treatments in comparison with the sham and control groups, but hypertrophic chondrocytes showed no obvious differences in comparison with the sham and control groups. Degenerating chondrocytes were reduced in experimental groups and revealed a decrease with doses of 150 and 500 mg/kg TiO_2_, suggesting a signifi cant difference.

## Discussion

The smaller size of the NPs causes a greater change in the specific embryonic areas, resulting in stronger reaction activity. These characteristics can reduce the stability of the cell membrane that leads to cell injury. It can also influence the activity and function of cells through interaction with macromolecules (9). There are great attention to toxicity of TiO_2_ NPs, but data are limited. Our results showed that the embyonic developmental processes were influenced by intraperitoneal injections of various doses of TiO_2_ NPs solutions. There are various studies regarding the potential neuronal uptake, translocation of inhaled particulates and pathological effects on the brian (10, 11). Researchers reported that anatase TiO_2_ NPs injected at the mouse abdominal cavity migrated anteriorly into the brain that resulted in the oxidative stress and injury of the brain, and subsequently disturbed the normal metabolism of neurochemicals (12). Our study showed that TiO_2_ NPs migrated into the uterus, affected the embryos, and caused the abortion. We observed a significant decrease in length of regions, suggesting that this reduction were dose-dependent. NPs (TiO_2_ or gold) are no longer distributed in the cytoplasm after being internalized by cells, so they are preferentially placed in mitochondria. When the mitochondria are invaded by NPs, the antioxidant defense ability may be changed (12). In another study investiated the biochemical parameters after daily injection of 5 nm anatase TiO_2_ NPs into the abdominal cavity of imprinting control region (ICR) mice for 14 days. The antioxidative responses observing in liver were reduced and hepatic lipids peroxide were increased in mice when using TiO_2_ NPs that suggested an oxidative attack (13). We worked on mice were injected on day 11 of gestation that was the critical point in limb bud development. We selected limb bud due to its variation in development and morphological changes. Results showed that TiO_2_ Nps caused different changes in chondrogenesis that led to a decrease in the number of mesenchymal cells that may be due to their differentiation into chondrocytes, suggesting a positive effect of TiO_2_ Nps on these cells. Furthermore, our findings revealed significant changes in resting, proliferating, and hypertrophic zones. Therefore TiO_2_ NPs accelerated the development of limb buds. Resting chondrocyte counts showed an increase as compared with the sham and control groups which means Nps prevented the differentiation of these cells in regions 1 and 3. The number of proliferating chondrocyte cells was insignificant in region 1 and 2, whereas surprisingly in region 3 that the mice were treated with 500 mg/kg TiO_2_ Nps, the number of cells showed a significant increase, suggesting that TiO_2_ Nps increased the proliferating chondrocyte cells by disrupting the cells cycle. So, the exposure dose is an important parameter in inducing toxicity. Genetically *fibroblast growth factors (FGFs)* play a crucial role in early step of chondrogenesis. These factors bind to their tyrosine kinase receptors and regulate proliferation and differentiation. Another tests on mice revealed requirements for *bone morphogenetic protein (BMP)* signaling pathways in multiple aspects of chondrogenesis such as proliferation and differentiation of cells and also demonstrated that progression of chondrocytes is controlled by the balance between signaling outputs from BMP and FGFs pathways (14, 15). Additionally *parathyroid hormone related protein (PTHrP)* along with Indian hedghog (Ihh) regulate cells from proliferation to hypertrophied stages by formation a negative feedback loop (16). Hypertrophic chondrocytes count in regions 1 and 2 showed a significant increase that may be due to differentiation of proliferating chondrocytes to hypertrophic chondrocytes that is in contrast to region 3, showing no obvious differences in comparison with the sham and control groups. Perichondrial cells in TiO_2_-treated groups showed a signiﬁcant increase in comparison with the control group because perichondrium is a type of irregular collagenous connective tissue that plays a role in growth and repair of cartilage. During aggregation of mesenchymal cells by SOX9 expression, cells in the center were committed to differentiate into cartilage, while cells in the periphery remained undifferentiated and appeared in form of perichondrium status (17). In a study that mice treated with the doses of 125 and 250 mg/kg BW anatase TiO_2_ NPs for consecutive 30 days displayed a decreas in body weight, seriously damaged liver function, as well as increased coefficients of the liver, kidney, spleen and thymus. It is very likely that liver function damage in mice is caused by higher anatase TiO_2_ NPs that is closely associated with the damage of haemostasis blood system and immune response because dose of 62.5 mg/kg TiO_2_ NPs has little influence on haemostasis blood system and immune response in mice (18). Another study showed high-dose of anatase TiO_2_ NPs (5 nm) through intraperitoneal injection could damage liver function (19). In our study, mesenchymal cells counts were signifi cantly higher in the 30 mg/kg TiO_2_ treatment that led to the increased level of resting condrocyts in regions 1 and 3, indicating that exposure dose have important role in toxicity. In an experiments rats were intra-tracheally instilled with 0.5, 5, or 50 mg/kg of 5, 21 and 50 nm TiO_2_ primary particles and their results showed that 5 and 21 nm TiO_2_ can induce pulmonary lesions when exposure dose is >5.0 mg/kg TiO_2_ particles. It is noted that if the exposure dose is ≥50 mg/kg, 5 nm TiO_2_ primary particles may suppress the phagocytotic ability of alveolar macrophages (AMs). Our results also confirmed the important roles of particle size and exposure dose. Further studies are needed to elucidate the underlying mechanisms and thier correlation with the physico-chemical properties of nano-TiO_2_ (20).

## Conclusion

TiO_2_ Nps could accelerate the development of limb buds in specific dose and particle sizes. We suggest a chondrogenic potential of TiO_2_ NPs which may be usefull in genetic abnormalities, although the toxicity of different concentrations of TiO_2_ NPs on organogenesis should be investigated further.
